# Protein and functional isoform levels and genetic variants of the BAFF and APRIL pathway components in systemic lupus erythematosus

**DOI:** 10.1038/s41598-022-15549-0

**Published:** 2022-07-02

**Authors:** Pilar Ortiz-Aljaro, Marco Antonio Montes-Cano, José-Raúl García-Lozano, Virginia Aquino, Rosario Carmona, Javier Perez-Florido, Francisco José García-Hernández, Joaquín Dopazo, María Francisca González-Escribano

**Affiliations:** 1 Department of Immunology, Hospital Universitario Virgen del Rocío (IBiS, CSIC, US), 41013 Seville, Spain; 2 Clinical Bioinformatics Area, Fundación Progreso y Salud (FPS), CDCA, Hospital Virgen del Rocio, 41013 Seville, Spain; 3 Bioinformatics in Rare Diseases (BiER), Centro de Investigación Biomédica en Red de Enfermedades Raras (CIBERER), FPS, Hospital Virgen del Rocío, Seville, Spain; 4 Computational Systems Medicine, Institute of Biomedicine of Seville (IBIS), Hospital Virgen del Rocio, Seville, Spain; 5 Department of Internal Medicine, Hospital Universitario Virgen del Rocío (IBiS, CSIC, US), 41013 Seville, Spain; 6 Functional Genomics Node, FPS/ELIXIR‐es, Hospital Virgen del Rocío, Seville, Spain

**Keywords:** Immunology, Biomarkers, Diseases, Medical research, Rheumatology

## Abstract

Systemic lupus erythematosus (SLE) is the prototype of an autoimmune disease. Belimumab, a monoclonal antibody targets BAFF, is the only biologic approved for SLE and active lupus nephritis. BAFF is a cytokine with a key-regulatory role in the B cell homeostasis, which acts by binding to three receptors: BAFF-R, TACI and BCMA. TACI and BCMA also bind APRIL. Many studies reported elevated soluble BAFF and APRIL levels in the sera of SLE patients, but other questions about the role of this system in the disease remain open. The study aimed to investigate the utility of the cytokine levels in serum and urine as biomarkers, the role of non-functional isoforms, and the association of gene variants with the disease. This case–control study includes a cohort (women, 18–60 years old) of 100 patients (48% with nephritis) and 100 healthy controls. We used ELISA assays to measure the cytokine concentrations in serum (sBAFF and sAPRIL) and urine (uBAFF and uAPRIL); TaqMan Gene Expression Assays to quantify the relative mRNA expression of ΔBAFF, βAPRIL, and εAPRIL, and next-generation sequencing to genotype the cytokine (*TNFSF13* and *TNFSF13B*) and receptor (*TNFRSF13B*, *TNFRSF17* and *TNFRSF13C*) genes. The statistical tests used were: Kruskal–Wallis (qualitative variables), the Spearman Rho coefficient (correlations), the Chi-square and SKAT (association of common and rare genetic variants, respectively). As expected, sBAFF and sAPRIL levels were higher in patients than in controls (p ≤ 0.001) but found differences between patient subgroups. sBAFF and sAPRIL significantly correlated only in patients with nephritis (r_s_ = 0.67, p ≤ 0.001) and βAPRIL levels were lower in patients with nephritis (p = 0.04), and ΔBAFF levels were lower in patients with dsDNA antibodies (p = 0.04). Rare variants of *TNFSF13 *and* TNFRSF13B *and* TNFSF13* p.Gly67Arg and *TNFRSF13B* p.Val220Ala were associated with SLE. Our study supports differences among SLE patient subgroups with diverse clinical features in the BAFF/APRIL pathway. In addition, it suggests the involvement of genetic variants in the susceptibility to the disease.

## Introduction

Systemic lupus erythematosus (SLE) [OMIM #152700] is the prototype of autoimmune disease. It is a chronic disorder characterized by autoantibodies (auAb) production, immune complexes deposit, and heterogeneous clinical manifestations. Patients present a wide range of symptoms, including photosensitive rashes, discoid lesions, arthritis/arthralgia, nephritis, heart and lung alterations, and central nervous system disorders. Approximately 50–60% of patients with SLE have lupus nephropathy. This manifestation, especially in its most severe presentation, significantly increases disease-related morbidity and mortality^1,2^.

Belimumab is the only biologic approved for SLE and active lupus nephritis treatment by numerous regulatory agencies, including EMA and FDA. It is a monoclonal antibody that targets against B cell-activating factor belonging to the TNF family (BAFF, also called TNFSF13B, Blys, αTNF4, THANK and TALL-1), is a cytokine with a key-regulatory role in the B cell homeostasis. BAFF controls the number of peripheral B cells by binding to three receptors: BAFF-R (also called TNFRSF13C and BR3), TACI (or TNFRSF13B) and BCMA (also named BCM and TNFRSF17). BAFF-R only binds to BAFF and is essential for the survival and maturation of immature B cells. TACI, which has a higher affinity for a BAFF-like protein called APRIL (a proliferation-inducing ligand, also called TNFSF13), is a pivotal receptor in T cell-independent B response, in control of B cell- compartment size, and the isotype switching. BCMA has an intermediate affinity to BAFF or APRIL and promotes plasma cell survival. The signalling cascade through BAFF-R and BCMA stimulates the proliferation of B lymphocytes and counteracts apoptosis^3^. BAFF and APRIL are type II transmembrane proteins produced by myeloid linage cells, although lymphoid cells, including B and activated T cells, can also generate them^4^. BAFF becomes into the active soluble form after cleavage at the furin-protease site. APRIL mainly is produced in the soluble form previously by processing in the Golgi apparatus before releasing^5,6^. BAFF and APRIL, the same as the rest of the members of the TNF family, assemble as homotrimers. They can also join as heterotrimers, and the BAFF-APRIL recombinant-heterotrimers are biologically active^7,8^. The receptors are type III transmembrane proteins with similar gene and protein structures^3^. Many studies reported elevated levels of functional soluble BAFF and APRIL in the sera of SLE patients. Nevertheless, results regarding clinical features, activity and correlation between the levels of both cytokines are conflicting^9,10^. Very few studies have investigated the usefulness of urinary detection as a biomarker^11,12^.

There are different isoforms of BAFF, products of alternative splicing, which could play a role in the pathogenesis of SLE. Especially interesting is ∆BAFF lacking exon 3, resulting in a shorter protein. Mouse ∆BAFF decreases bioactivity by associating with the complete forms become inactive heterotrimers. Similar events occur in APRIL, although with a non-well established functional impact. For instance, similarly to ∆BAFF, the βAPRIL isoform lacks an alternate in-frame exon in the central coding region, resulting in a shorter protein than canonical. In addition, no detection of sAPRIL in the supernatant of cells transfected with the β isoform has been reported^13^. εAPRIL isoform lacks exons 2 and 3 and represents a non-coding variant because the transcript is a candidate for nonsense-mediated mRNA decay.

SLE is a complex disease with a genetic basis and almost 30 genetic regions validated as predisposing to the disease^14^*. TNFSF13B* (HGNC:11929) 13q33.3 encodes human BAFF, *TNFSF13* (HGNC:11928) 17p13 encodes APRIL, *TNFRSF13C* (HGNC:17755) 22q13.2 encodes BAFF-R, *TNFRSF13B* (HGNC:18153) 17p11 encodes TACI, and *TNFRSF17* (HGNC:11913) 16p13 encodes BCMA^3^. Several studies reported the association of polymorphism in the genes of these cytokines and their receptors with autoimmune diseases, immunodeficiencies and cancer^15^.

To assess the involvement of the BAFF/APRIL system and its receptors in the development and clinical course of SLE, we performed a study evaluating the levels of the proteins in serum and urine and the role of non-functional isoforms, and the association of variants in cytokine and receptor genes.

## Materials and methods

### Study cohort

Table 1 displays the features of the cohort. This study includes a cohort of 100 SLE-unrelated patients (women between 18 and 60 years old; mean age 43.8 years; 48 with lupus nephritis inactive at the sampling time) who fulfilled the 1997–2012 Systemic Lupus International Collaborating Clinics/American College of Rheumatology classification criteria for SLE^16,17^. The control group consisted of 100 healthy unrelated women ethnically matched (mean age 37.8 years). All the subjects are Spanish Caucasians recruited from Hospital Universitario Virgen del Rocío, Seville, Spain. The local ethics committee of the hospital approved the study, and all participants gave written informed consent before inclusion. We collected serum, urine and anticoagulated blood samples from each participant. Supplementary Fig. 1 exhibits the number of available specimen samples for each group in each assay.

**Table 1:** Patient and control features.

	Patients		Controls
	Without nephritis N = 52	With nephritis N = 48	N = 100
Age (mean ± SD)	43.5 ± 8.4	42.5 ± 11.8	36.8 ± 11.5
**Autoantibodies**			
ANA positive	79%	91%	0%
Anti dsDNA positive	28%	25%	1%
Nephritis		48%	
Without biopsy		47%	
**Biopsy grade**			
II		9%	
III		7%	
IV		36%	
V		2%	
**Activity (SLEDAI)**			
Inactive (0–1.99)	66%	61%	
Mild (2–3.99)	21%	32%	
Moderate (4–7.99)	13%	5%	
Severe (more than 8)	0%	2%	
**Treatment**			
Hydroxychloroquine monotherapy	72%	42%	
Hydroxychloroquine in combination	2%	22%	
Others	4%	7%	
Without any treatment	21%	29%	

### Quantification of the serum and urine levels of BAFF and APRIL

We used Enzyme-Linked Immunosorbent Assays (ELISA) to quantify the cytokine levels in serum and urine. In the case of BAFF (TNFSF13B), human in vitro Simple Step ELISA^®^ (ab188391-BAFF; Abcam, 330 Cambridge Science Park, Cambridge CB4 0FL, UK) was used according to the manufacturer's recommendations. Briefly, this system employed two antibodies, an affinity tag-labelled to capture and a reporter conjugated as the detector. The entire complex (capture antibody + analyte + detector antibody) is immobilized via immunoaffinity using an anti-tag antibody coated onto the well, testing 100 μL of each specimen (serum or urine) in each assay. The concentration, expressed in ng/mL, is calculated by optical density (OD) extrapolation with a standard curve (range 0–5 and the minimal detectable amount, MDA = 0.0127 ng/mL). APRIL (TNFSF13) Human in vitro ELISA kit (ab119505; Abcam) was employed according to the manufacturer's recommendations to quantify APRIL. This system uses APRIL specific antibodies on pre-coated plates to test 50 μL of each specimen (serum or urine) using an anti-APRIL biotinylated antibody as a second antibody. The OD is directly proportional to the APRIL amount captured on the plate. The concentration, expressed in ng/mL, is calculated by OD extrapolation with a standard curve (range 0–5 and MDA = 0.40 ng/mL).

### Antinuclear antibodies screening and anti-dsDNA levels

To investigate the presence of antinuclear antibodies (ANA), used ANA screen ELISA (Meridian Bioscience). To determine the specificities in positive sera, used the routinary method applied in the laboratory (EUROLINE ANA Profile 3 IgG kit, Euroimmun).

To quantify anti-dsDNA antibody levels, tested a sample of 9 μL of serum from each participant with the Elia dsDNA test (Thermo Fisher Scientific) using a β- Galactosidase-conjugated secondary anti-IgG antibody. Anti-dsDNA antibody levels assignment, by comparing the fluorescence signals of the samples and calibrators (with known concentrations), and expressing the results in IU/mL (range 0.5–379 IU/mL) and classifying samples as neg < 10 IU/mL, doubtful 10–15 IU/mL and positive > 15 IU/mL.

### Quantification of mRNA levels of the BAFF and APRIL isoforms

For ΔBAFF and β and εAPRIL mRNA levels quantification, 10^7^ peripheral blood mononuclear cells isolated obtained by density gradient were used to extract the total RNA with QIAmp RNA Mini Kits (Qiagen, Barcelona, Spain). We quantify total RNA by measurement of the OD260 and verify the integrity by electrophoresis and 260/280 nm absorption ratio. Concentrations ≥ 30 ng/μL and A260/280 absorbance ratios > 1.8 were considered acceptable, and stored samples were at − 80 °C until use.

The cDNA synthesis was performed by reverse-transcription using one µg of total RNA and random primers and the Superscript™ FirstStrand Synthesis System for RT-PCR (Thermo Fisher Scientific, Waltham, MA). Quantification of mRNA was performed by real-time PCR on a LightCycler 480 (Roche, Barcelona, Spain) using TaqMan Gene Expression Assays (ThermoFisher Scientific). (Supplementary Fig. 2). We tested the samples in duplicates, including a tube without any template (negative control) in each run. Data were analyzed with the LightCycler 4.05 software using the Calibrator Normalized Relative Quantification module with the efficiency correction method. A pool of cDNA from control samples was used as a calibrator to set a relative value of 1. Only those values within the linear area of the standard curves were acceptable. Samples with Cp values > 35 and duplicates with a standard deviation of Cp > 0.3 were re-tested. The relative mRNA levels are expressed as the ratio of mRNA of the target isoform (ΔBAFF and β and εAPRIL) and the reference isoforms of the corresponding gene (BAFF isoform-1 and APRIL isoforms-α + γ) and normalized to the calibrator expression ratio.

### Genotyping of the genes of the cytokines and their receptors

Following the manufacturer's instructions, we used peripheral blood samples to obtain DNA with QIamp DNA mini kits (Qiagen, Barcelona, Spain). Samples were stored at − 20 °C until used. DNA concentrations were quantified in a Qubit^®^ 3.0 fluorometer and diluted to 0.67 ng/µl.

We genotype DNA samples by next-generation sequencing (NGS) in a custom-designed primer panel (AmpliSeq™ software, Ion Torrent, Thermo Fisher Scientific, Waltham, MA). This panel targeted all the coding regions and flanking intronic sequences of the five genes included in the study: *TNFSF13B *(*NG_029524*), *TNFSF13 *(*NG_029949*), *TNFRSF13B* (*NG_007281*)*, TNFRSF13C *(*NG_007579*) and *TNFRSF17* (*NM_052945.4*). Amplifications and barcoding libraries were carried out with Ion AmpliSeq™ Kit for Chef DL8 (Thermo Fisher Scientific, Waltham, MA) according to the manufacturer's recommendations. To quantify libraries we used qPCR or with Qubit^®^ 3.0 Fluorometer, diluting samples to a final concentration of 40 pM and using them for template preparation with the Ion 510™ and Ion 520™ and Ion 530™ Kit on the Ion Chef™ System. Sequencing templates were loaded in Ion 520™ Chips and sequenced in an Ion S5™ Primer Sequencer. The total of amplicons had a coverage greater than 30X. We used Ion Reporter™ Software v12.2 to call the variants and aligned the readings to the human genome reference, hg19. We visually reviewed those variants reported by the software to confirm the correct reading alignments with the Integrative Genomics Viewer IGV v2.3.68, Broad Institute^18^.

### Statistical analysis

The variance test (Bartlett Test) had p values < 0.05 in all the cases, so we used nonparametric methods (Kruskal–Wallis) for qualitative variables comparisons (patients and controls or patients with different features) (Epi Info v7.2.4, https://CRAN.R-project.org/package=Epi). Quantitative values display as median, interquartile range (IQR, Q3–Q1). The variable-correlation study includes only individuals without missing data, using the Spearman Rho coefficient because data exhibits a non-linear association between variables and contains outliers (https://www.socscistatistics.com/tests/spearman/). When significant, the r_s_-values 0–0.20 were considered very weak correlation, 0.20–0.39 weak correlation, 0.40–0.69 moderate correlation, 0.70–0.89 strong correlation, and 0.90–1.00 very strong correlation. In all the comparisons, the p-values < 0.05 were considered significant.

In the association study, minor allele frequencies (MAF) of genetic variants found in our study (91 patients and 91 controls) were obtained through the Cellbase database^19^ using the 1000 Genomes Project data source. This way, variants were classified as commons (MAF ≥ 0.05), rares (MAF < 0.05) or undetected (MAF = 0). We catalogued all the variants according to the American College of Medical Genetics and Genomics (ACMG) criteria using Varsome (https://varsome.com/) as Benign (B), Likely Benign (LB), Variant of Uncertain Significance (VUS), likely pathogenic (LP), and pathogenic (P). Common and rare variants were treated separately in the case–control study. We used the Chi-square to evaluate the association of the common variants, and p-values were adjusted using the false discovery rate (FDRadj.pval). To analyze the association of the rare variants, we used Vartools, a module of the Zhbannikov software (http://izhbannikov.github.io/vartools/) and applied three different strategies. The first included all the rare variants in each gene (MAF ≤ 0.05 in 1 KG-ALL). The second, those rare variants with possible functional relevance (MAF ≤ 0.05 in 1 KG-ALL annotated as missense and frameshift variants). Lastly, the third included those rare variants with a probable clinical significance (MAF ≤ 0.05 catalogued as VUS, LP or P according to the *ACMG* criteria). The analysis with the SNP-set Kernel Association Test (SKAT)^20^ includes those genes with at least two rare variants that meet the filter criteria. A p-value < 0.05 was considered significant.

### Ethical approval

The Hospitales Universitarios Virgen Macarena and Virgen del Rocio’s ethical committee approved this study (peiba_DictamenFavorable2019114215856). The authors declared that all methods were carried out following relevant guidelines and regulations.

## Results

Table 1 displays the characteristics of the cohort. Patients were selected so that the proportion of those with and without nephritis was approximately 1:1. Regarding the autoantibodies, 85% of patients had a positive result in the ANA test in the serum obtained for this study, and only 27% had anti-dsDNA antibodies in the same serum. The most common autoantibodies specificities were Ro/La (27%) and Sm/RNP (19%). Most of the patients had a non-active disease and received treatment with hydroxychloroquine in monotherapy or combination.

### Quantification of the serum and urine levels of BAFF and APRIL

**Figure 1:**
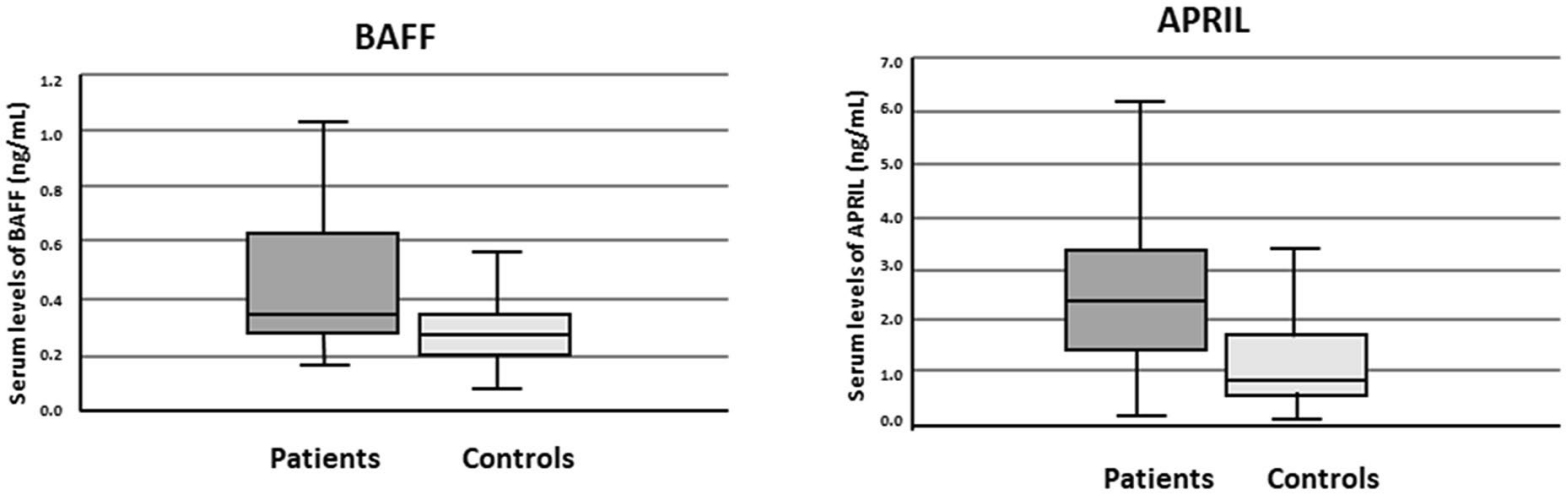
Median levels of sBAFF and sAPRIL in patients and controls.

Median levels of BAFF and APRIL in serum (sBAFF and sAPRIL) were higher in patients than in controls (p < 0.001 in both cases) (Fig. 1). Both serum cytokine levels significantly correlated in patients with nephritis but not in patients without or in controls (Table 2). There were no significant differences in the median levels of both cytokines between patients with and without nephritis (Supplementary Fig. 3), even after grouping by their histologic diagnosis of lupus nephritis II vs. III + IV + V grades**.** We also found no significant differences in the cytokine serum levels when comparing patients with and without anti-dsDNA antibodies or patients with active and inactive disease. (Supplementary Figs. 4 and 5).

**Table 2:** Correlation between different pairs of markers in patients and controls.

Correlation	Patients				Controls	
	Without nephritis N = 32		With nephritis N = 29		N = 51	
	p	r_s_	p	r_s_	p	r_s_
sBAFF/sAPRIL	> 0.05		**< 0.01**	**0.67**	> 0.05	
sAPRIL/uAPRIL	> 0.05		> 0.05		**< 0.01**	**0.42**
βAPRIL/εAPRIL	**< 0.01**	**0.64**	**< 0.01**	**0.61**	**< 0.01**	**0.36**
βAPRIL/sAPRIL	> 0.05		> 0.05		> 0.05	
εAPRIL/sAPRIL	> 0.05		> 0.05		**0.02**	**0.33**
ΔBAFF/sBAFF	> 0.05		> 0.05		> 0.05	

The correlation analysis was performed only with those individuals with available data in all the assays.

Only the r_s_ values of those statistically significant correlations (p-values < 0.05) are displayed (shown in bold).

Only two patients (with dsDNA antibodies, without nephritis and with a mild activity) had sBAFF concentration values over 2.0 ng/mL. There were no significant differences in the features of the groups of patients with sAPRIL values greater or less than 4.0 (N = 15: 3, 20%, with anti-dsDNA antibodies, 7, 47% with nephritis, 5, 33% with active disease vs. N = 72: 21, 29% with anti-dsDNA antibodies, 35, 49% with nephritis, 26, 36% with active disease).

Regarding the urine, only one individual (patient) had detectable uBAFF levels (> 0.0127 ng/mL), whereas 61 samples had detectable uAPRIL (> 0.40 ng/mL). The percentage of individuals with detectable uAPRIL was lower in patients than in controls (patients: 18 out of 82, 22% and healthy controls: 43 out of 86, 50%; p = 0.0002, OR 0.28, 95% CI 0.14–0.55). sAPRIL and uAPRIL levels correlated in controls but did not in patients (Table 2).

### Quantification of mRNA levels of the ΔBAFF and β and εAPRIL isoforms

**Figure 2:**
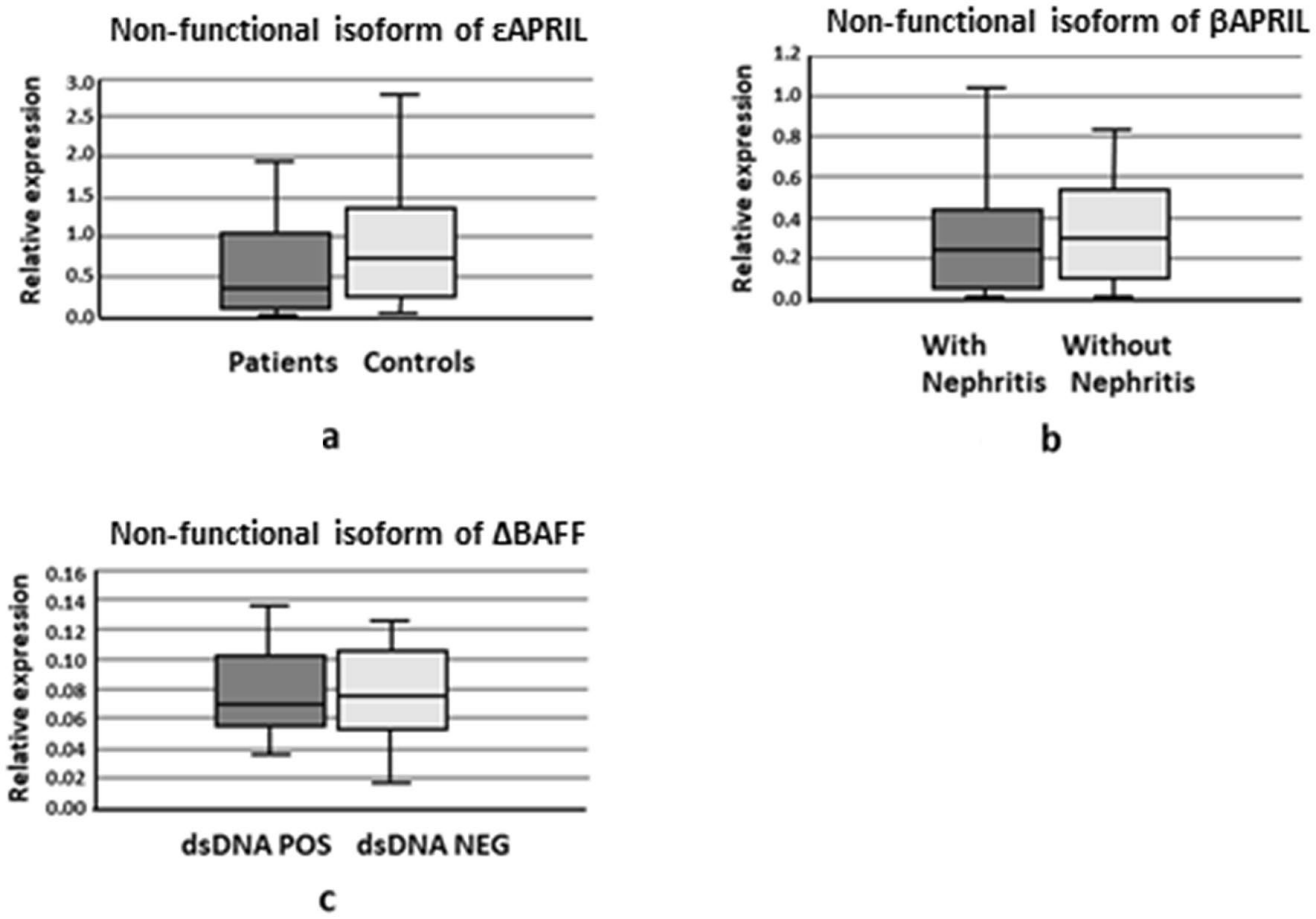
Median of the relative expression of non-functional isoforms of BAFF and APRIL. (**a**) Median of the relative expression εAPRIL/α+γ− in patients and controls. (**b**) Median of the relative expression βAPRIL/α+γ in patients with and without nephritis. (**c**) Median of the relative expression ΔBAFF/functional isoforms in patients with and without dsDNA antibodies.

Concerning the relative expression of non-functional isoforms in patients and controls, there were no significant differences in the cases of ΔBAFF and βAPRIL However, the εAPRIL median relative expression was lower in patients than in controls (Fig. 2a). For patient subgroups, the relative expression of βAPRIL was lower in patients with nephritis than in patients without (Fig. 2b), and the relative levels of ΔBAFF were lower in patients with dsDNA antibodies than in patients without (Fig. 2c). The rest of the comparisons were not statistically significant (data not shown).

The relative expression levels of β and εAPRIL correlated in patients and controls, whereas the relative expression levels of εAPRIL with levels of sAPRIL only correlated in controls. Lastly, the relative expression levels of the βAPRIL and ΔBAFF did not correlate with the serum levels of the corresponding protein in any group (Table 2).

### Association study of the genes of the cytokines and their receptors

Supplementary Table 1 displayed detailed information on the 52 variants found in the five genes included in the study. Table 3 exhibits the number of variants found in each gene and their classification according to frequencies in the 1000 Genome all population Database and the ACMG criteria. Most of the variants are single nucleotide variants (SNV, 46, 88%) catalogued as rare variants (MAF < 0.05 in the 1000 Genome all population Database) (Supplementary Fig. 6).

**Table 3:** Classification of genetic variants according to their MAF and their ACMG pathogenicity criteria.

Gene	Frequencies of variants in 100 genome all populations database														
	MAF > 0.05					0 < MAF < 0.05					MAF = 0				
	B	LB	VUS	LP	P	B	LB	VUS	LP	P	B	LB	VUS	LP	P
*TNFSF13B* 6 variants	1						2	1				1	1		
*TNFSF13* 12 variants	3					2	4					2	1		
*TNFRSF13B* 21 variants	5	1	1			3	1	1	2	1	1		4		1
*TNFRSF13C* 9 variants	1		2			3	1					1	1		
*TN*F*RSF17* 4 variants	2					1	1								
Total 52 variants	12	1	3			9	9	2	2	1	1	4	7		1

*ACMG* American College of Medical Genetics and Genomics, *MAF* minor allele frequency, *B* benign, *LB* likely benign, *VUS* variant of uncertain significance, *LP* likely pathogenic, *P* pathogenic.

From a total of 16 common variants (MAF > 0.05 in the 1000 Genome all population Database), only in one case, *TNFSF13* p.Gly67Arg (rs11552708), the distribution was significantly different in patients and controls (MAF in patients 0.10, MAF in controls 0.03 p = 0.014, OR 3.21 95% CI 1.25–9.02), although the FDRadj.pval > 0.05 (Table 4).

**Table 4:** Association of the gene common variants with SLE.

Gene	Variant	ID	ACMG	Number of alleles		MAF	
				Patients N = 182	Controls N = 182	Patients	Controls
*TNFSF13B*	c.340-45C>G	rs56124946	B	8	3	0.04	0.02
*TNFSF13*	p.Gly67Arg^1^	rs11552708	B	18	6	0.10	0.03
	p.Asn96Ser^2^	rs3803800	B	143	142	0.21	0.22
	c.505-20_505-18delACA	rs58840546	B	12	11	0.07	0.06
*TNFRSF13B*	c.*173G>A	rs56153623	B	71	66	0.39	0.36
	p.Ser277Ser	rs11078355	B	79	77	0.43	0.42
	p.Pro251Leu	rs34562254	B	21	15	0.12	0.08
	c.632-60T>C	rs11652811	B	60	60	0.33	0.33
	c.445+144A>G	rs4517836	B	57	56	0.31	0.31
	c.445+25A>C	rs2274892	VUS	84	76	0.46	0.42
	p.Thr27Thr	rs8072293	LB	119	118	0.65	0.65
*TNFRSF13C*	c.368-33T>C	rs5996087	VUS	16	14	0.09	0.08
	c.367+89G>C	rs73165134	VUS	16	11	0.09	0.06
	c.136+140G>A	rs150150552	B	0	3	0.00	0.02
*TNFRSF17*	p.Thr159Thr	rs2017662	B	11	13	0.06	0.07
	p.Thr175Thr	rs2071336	B	6	9	0.03	0.05

*ACMG* American College of Medical Genetics and Genomics. ^1^All p-values > 0.05 except TNFSF13 p.Gly67Arg p value = 0.014 FDRadj.pval > 0.05. ^2^MAF (Minor Allele Frequency) correspond to the reference that is the minor allele in EUR.

Regarding the rare variants, *TNFRSF13B* Val220Ala (considered B) was the only associated according to an individual test (MAF in patients 0.0 vs. 0.02 in controls, p = 0.04. FDRadj.pval > 0.05). Concerning the analysis of rare variants at the gene level, *TNFRSF13B* was associated with SLE using SKAT with all the three filters applied: including all the variants with MAF < 0.05 (p = 0.03), restricting the analysis to those rare variants missense or frameshift (p = 0.006) and including only rare variants VUS, LP and P (p = 0.01). The *TNFRSF13B* rare variant number was lower in patients (20/182 alleles) than in controls (37/182 alleles). Also, the number of individuals with *TNFRSF13B* rare variants was lesser in patients (15/91, 17%) than in controls (24/91, 26%), although the difference was not statistically significant (p = 0.052). *TNFSF13B* was significantly associated with SKAT with rare variants VUS, LP and P (p = 0.04) though it did not reach significance with all the rare variants included in the test (p = 0.08). For the remaining genes, statistical significance was lacking. *TNFSF13* was near statistical significance with SKAT in the filter with all the rare variants included in the test (p = 0.057), but this gene did not meet the inclusion criteria with the other two filters (Table 5).

**Table 5:** Analysis of the association of rare variants in the five genes included in the study.

Gene	Number of variants that meet criteria	p-values including all the variants MAF < 0.05
		^**1**^**SKAT**
*TNFSF13B*	5	0.09
*TNFSF13*	9	0.06
*TNFRSF13B*	14	**0.03**
*TNFRSF13C*	6	0.14
*TNFRSF17*	2	0.33

Gene	Number of variants that meet criteria	p-values including those variants MAF < 0.05 missense or frameshift
*TNFSF13B*	2	0.15
*TNFRSF13B*	8	**0.006**
*TNFRSF13C*	3	0.18

Gene	Number of variants that meet criteria	p-values including those variants MAF < 0.05 VUS, LP, P
*TNFSF13B*	2	**0.04**
*TNFRSF13B*	9	**0.01**

^1^SNP-set (Sequence) Kernel Association Test Method (SKAT, Asymptotic p-value). This test analyzes the association of rare variants (Minor Allele Frequency, MAF < 0.05) grouped by the gene. The p-values < 0.05 were considered significant (shown in bold).

*VUS* variant of uncertain significance, *LP* likely pathogenic, *P* pathogenic.

Only the genes that met the evaluation criteria are displayed. The rest of the genes could not be evaluated with the corresponding filter because they did not have at least two rare variants that meet the condition.

## Discussion

In agreement with most of those previously published, our study reports higher levels of sBAFF and sAPRIL in female SLE patients than in healthy controls ethnically, gender and age-matched (revised in^9,10^). Our results suggest no influence of the serum levels of these cytokines with the dsDNA antibodies or the activity. Several studies reported discrepancies regarding the relationship between the concentration of these cytokines, the clinical features and the activity score^9,10^. Characteristics of the different cohorts and the index of activity used may influence the results. In this sense, our cohort consisted of patients with a low activity index. Also, the number of patients with and without nephritis is equivalent, but it is unbalanced regarding the dsDNA antibodies and the activity. These characteristics could influence the relatively low serum concentration of both cytokines found. In the case of sBAFF, only two patients had concentrations over those established as an independent prognostic factor for flares (2 ng/mL)^21^. Regarding sAPRIL, patients with sAPRIL values above and below the cut-off point reported as a predictor of the resistance to treatment (4.0 ng/mL)^22^ were not significantly different concerning the clinical variables analyzed.

A previously investigated question that has reported contradictory results is the coexistence of both molecules in the serum of SLE patients^23–25^. Our results strongly support the coexistence of both cytokines in the serum of patients with nephritis with a moderate correlation. This correlation in the subgroup of patients with nephritis may be due to parallel changes in the levels of both cytokines, but also to the fact that the levels of heterotrimers are higher in this subgroup (because both BAFF and APRIL ELISA assays detect heterotrimers) Previous studies reported higher levels of heterotrimers in patients with SLE compared with healthy control and patients with rheumatoid arthritis^7,8^. The restriction to patients with nephritis could explain the contradictory results on the coexistence of both molecules in SLE patient sera and be relevant in the treatment options. Thus, Belimumab, which blocks BAFF without neutralizing APRIL, has a minimal inhibitory activity on heterotrimers BAFF_2_APRIL_1_ and no activity in BAFF_1_APRIL_2_^26,27^. Theoretically, patients with the most elevated levels of both cytokines or heterotrimers would respond worse to the Belimumab treatment. There are studies which are reported differences in the kinetic sBAFF and sAPRIL in immunotherapy without BAFF blockade. The sAPRIL levels decreased after treatment predicting the response in patients with proliferative lupus nephritis. sBAFF levels under 1.5 ng/mL at baseline are a good predictor of response in patients with lupus nephritis but remain unchanged afterwards. So, even with conventional immunotherapy, sBAFF and sAPRIL could be used as biomarkers predictors of response^28^. Further studies are necessary to establish how the coexistence of both cytokines or levels of heterotrimers influences the response to treatments.

Another remaining question is about urinary excretion of BAFF and APRIL, proposed as biomarkers of SLE. The urinary excretion of BAFF seems to be an uncommon event, whereas the urinary excretion of APRIL may be a physiological process because it was detected often in controls. Although these results may seem surprising, similar findings have previously been published^29^. Therefore, our results do not support the utility of these urinary cytokines as biomarkers of SLE. It hypothesized that the urinary excretion of the cytokines could reduce their serum levels. In our study, the correlation between uAPRIL and sAPRIL levels in controls is robust, although moderate, and lacking in patients. The meaning of the lack of correlation between uAPRIL and sAPRIL levels in patients could be related to the disease though this result would need replication.

BAFF and APRIL, as a result of alternative splicing events, have different mature forms of mRNA. The non-functional isoforms such as ΔBAFF, βAPRIL and εAPRIL may regulate the active form of the protein, but their functional impact is not well characterized yet. To our knowledge, there are no previous assessments of the role of the non-functional isoforms of these cytokines in SLE. The mRNA simultaneous transcription of the two isoforms of APRIL may be a physiological process because their levels correlate in patients and controls. εAPRIL, underproduced in the patient group, is a non-coding variant which could have regulatory functions^30^. The positive correlation between εAPRIL and sAPRIL in healthy controls that is lacking in patients could indicate that, physiologically, the production of this isoform is a mechanism of response to elevated protein levels. This control mechanism may be disturbed in patients, the group in which this isoform is underproduced. Our results do not support any influence of βAPRIL and ΔBAFF on the serum protein levels because there was no correlation in patient or control groups. In addition, βAPRIL was underproduced in patients with nephritis and ΔBAFF in patients with anti-dsDNA antibodies, suggesting an influence of the regulatory activity of these isoforms in some patient subgroups.

Many studies investigated the genetic factors related to SLE by comparing the distribution of single nucleotide polymorphisms (SNPs) in patients and controls. The high-throughput genetic studies (such as Genome-Wide Association Studies, GWAS) have been very informative in SLE and other polygenic diseases. Nevertheless, GWAS are not approaches designed to detect rare variants, which are lost even with imputation processes because their extremely-low frequency. The NGS permits the analysis of the common and the rare variants. This fine-mapping approach could help solve the missing heritability problem and validate therapeutic targets. Concerning the common variants included in our study, the results suggest an association of the *TNFSF13* p.Gly67Arg in our population (risk variant Arg67), although the p-value became non-significant after correction. Several studies reported an association of this position with SLE in Japanese and, to a lesser extent, in African-Americans and Hispanics (risk variant Gly67), but not in American white people^31–33^. *TNFSF13* p.Gly67Arg is a B variant according to the ACMG criteria, and its functional significance remains unknown. Altogether, results suggest an association with the region but rule out p.Gly67Arg as the causal variant. In agreement with most of the previously published studies^34–37^, there were no common variants associated with SLE in the other four genes inside the region included in our study^34–38^, although the association with other diseases has been described^39–42^. Regarding the individual analysis of rare variants*, TNFRSF13B* p.Val220Ala was associated with the disease, being Ala220 lesser common in patients. This variant is considered B, but it could disturb the stability centres of the protein^42^. In any case, the individual associations study of rare variants in complex diseases has difficulties because of the lack of statistical power. To get around this question, specific statistical methods: grouping variants and checking the association of the entire gene, have been designed. In the present study, *TNFRSF13B* was associated with the disease independently of the strategy used. Variants in *TNFRSF13B* have been associated with antibody deficiency, finding a high frequency of variants in patients with common variable immunodeficiency and IgA deficiency^43^. But, the accumulation of variants in *TNFRSF13B* could confer some evolutionary advantage since the gene has an unexpected diversity and the IgA deficiency is often asymptomatic^44^. In this sense, the number of rare variants in *TNFRSF13B* in our cohort of patients was lower than in controls. The other gene with associated rare variants was *TNFSF13B,* but only in the analysis restricted to variants VUS + LP + P. Previous studies reported a rare INDEL variant GCTGT>A located outside the region included in our study (in the 3´UTR region of *TNFSF13B* gene) as associated with SLE and other autoimmune diseases^45^. Association was not detected for the remaining genes, although their study has more limitations and, in general, our findings with rare variants need replication in other cohorts.

In conclusion, our study supports differences among patient subgroups in the coexistence of both cytokines or the levels of heterotrimers. It suggests a role of the non-functional isoforms that may also be related to clinical features. In addition, it supports the involvement of rare variants in *TNFSF13B* and *TNFRSF13B* in the disease.

## Supplementary Information

Supplementary Information.

## Data Availability

All data relevant to the study are included in the article or uploaded as supplementary information. This study is registered in ClinicalTrials.gov (https://register.clinicaltrials.gov/prs) NCT 03919643 (Initial release 02/14/2019). Further data can be made available upon request mariaf.gonzalez.sspa@juntadeandalucia.es.

## Abbreviations

ACMG: American College of Medical Genetics and Genomics
ANA: Antinuclear antibodies
APRIL: A proliferation-inducing ligand
B: Benign (genetic variant)
BAFF: B cell-activating factor belonging to the TNF family
BAFF-R: BAFF receptor
BCMA: B-cell maturation antigen
LB: Likely benign (genetic variant)
LP: Likely pathogenic (genetic variant)
MAF: Minor allele frequency
NGS: Next-generation sequencing
P: Pathogenic (genetic variant)
SLE: Systemic lupus erythematosus
SKAT: SNP-set (Sequence) Kernel Association Test Method
TACI: Transmembrane activator and CAML interactor
TNFRSF: Tumor necrosis factor receptor superfamily
TNFSF: Tumor necrosis factor superfamily
VUS: Variant of Uncertain Significance (genetic variant)
