# Supplementary material for: Protein and functional isoform levels and genetic variants of the BAFF and APRIL pathway components in systemic lupus erythematosus
Source: Sci Rep. 2022 Jul 2;12:11219. doi: 10.1038/s41598-022-15549-0 (PMC9250527; doi:10.1038/s41598-022-15549-0)
Supplement: Supplementary file 1 — Supplementary Information. [file 41598_2022_15549_MOESM1_ESM.pdf]

SI. Figure 1. Flowchart with number of samples collected from each specimen and available in each assay

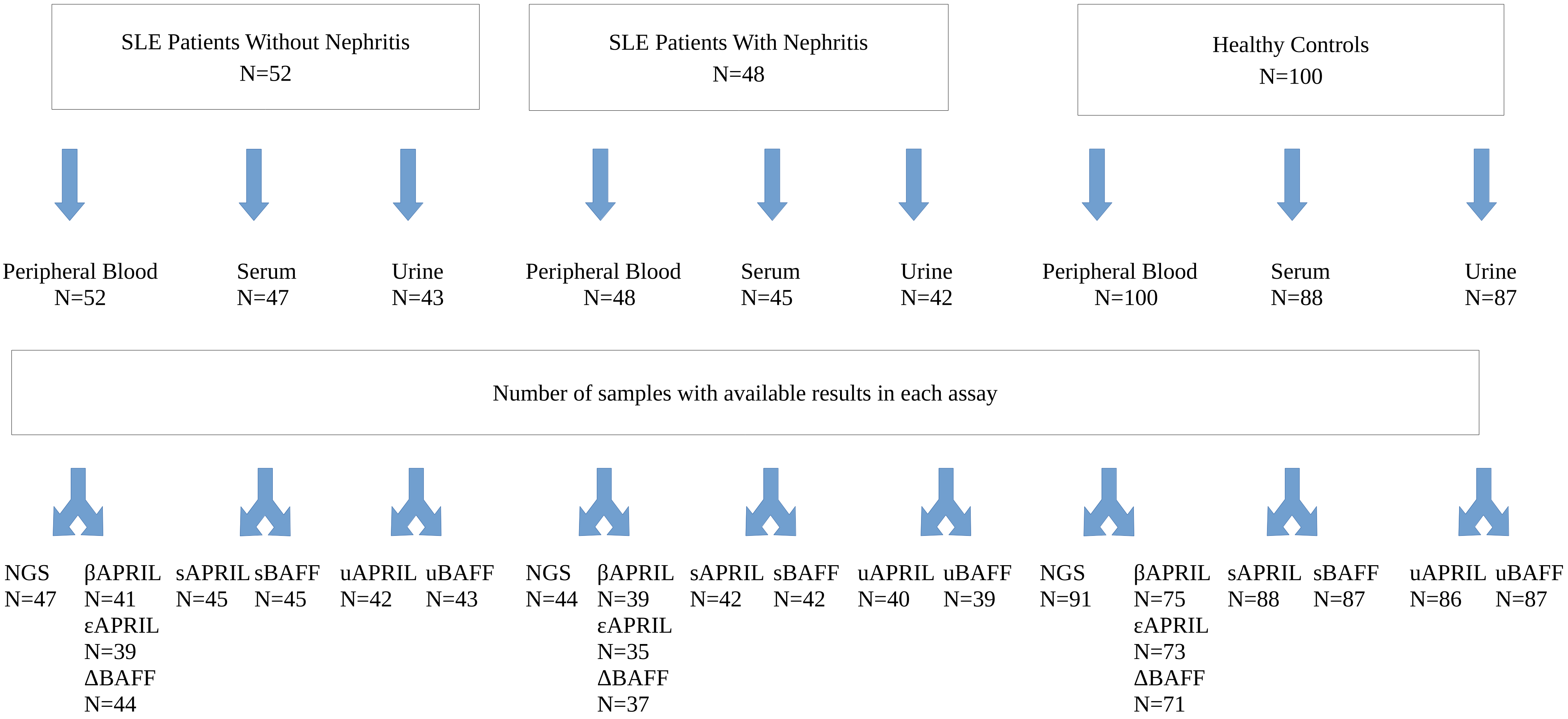

DNA and RNA were extracted from peripheral blood. DNA was used in Next Generation Sequencing (NGS) to study the variants in the genes of the cytokines and their receptor. RNA was used to quantify different functional isoforms of APRIL and BAFF.

Serum and urine samples were used to quantify the cytokines.

The number of individuals included in the correlation analysis (with available data in all the assays) was: patients without nephritis N=32, with nephritis N=29, healthy controls N=51

SI. Figure 2. TaqMan Gene Expression Assays for ΔBAFF and β and εAPRIL

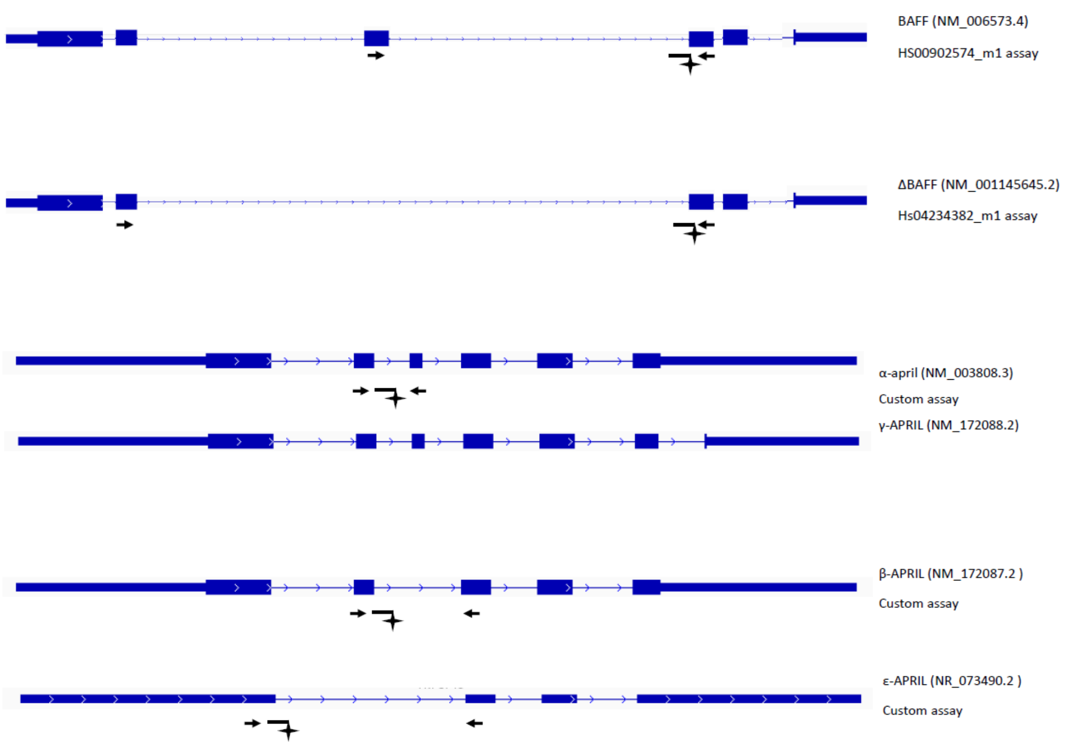

Concerning BAFF, the mRNA expression levels of the isoform ΔBAFF (mRNA-expression of isoform 2, NM_001145645.2) were relativized to the isoform 1 (NM_006573.4) used as reference. In the case of APRIL, the mRNA expression levels of the isoforms β and ε (βAPRIL, NM_172087.2 and εAPRIL, NR_073490.2) were referenced to the functional isoforms α and γ (αAPRIL, NM_003808.3 and γAPRIL, NM_172088.2). The figure shows the location of the primers and probes in each assay, designed at the junctions of the corresponding exons (BAFF exons 3-4, ΔBAFF exons 2-4, α and γAPRIL exons 2-3, βAPRIL exon 2-4 and εAPRIL exons 1-3).

SI Figure 3. Levels of sBAFF (2a) and sAPRIL (2b) in patients with and without nephritis.

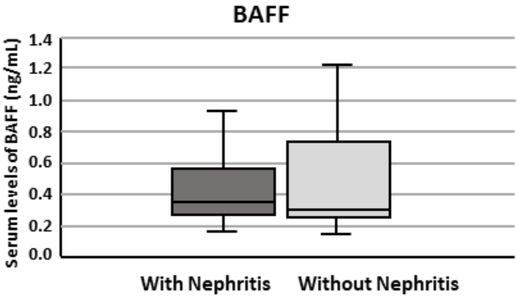

SI Figure 3a

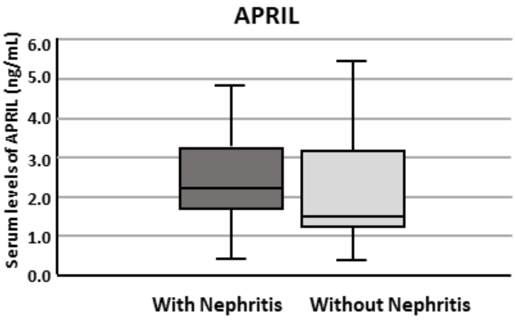

SI Figure 3b

Median sBAFF: Patients with nephritis 0.34, IQR 0.28 Vs. patients without 0.34, IQR 0.47; $p>0.05$

Median sAPRIL: Patients with nephritis 2.21, IQR 1.57 Vs. patients without 1.51, IQR 2.0; $p>0.05$

SI Figure 4. Levels of sBAFF (3a) and APRIL (3b) in patients with and without dsDNA antibodies.

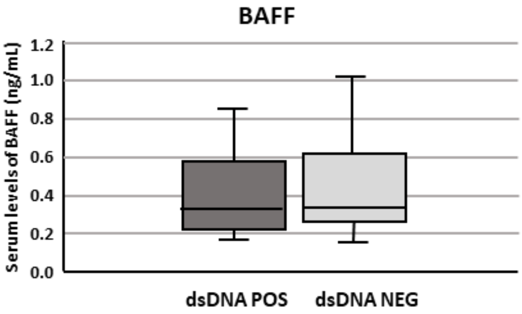

SI Figure 4a

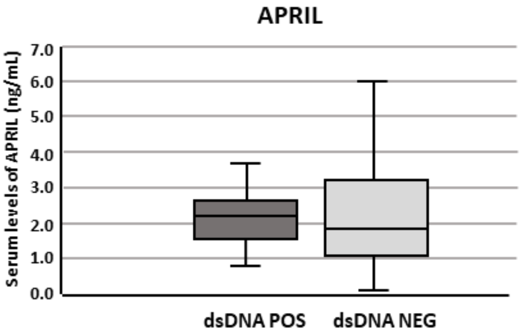

SI Figure 4b

Median sBAFF patients with dsDNA antibodies 0.34, IQR 0.33 Vs. patients without 0.34, IQR 0.36; p>0.05

Median sAPRIL in patients with dsDNA antibodies 2.19, IQR 0.96 Vs. patients without 1.93, IQR 2.1; p>0.05

SI Figure 5. Levels sBAFF (4a) and APRIL (4b) in patients stratified according their activity index score (SLEDAI).

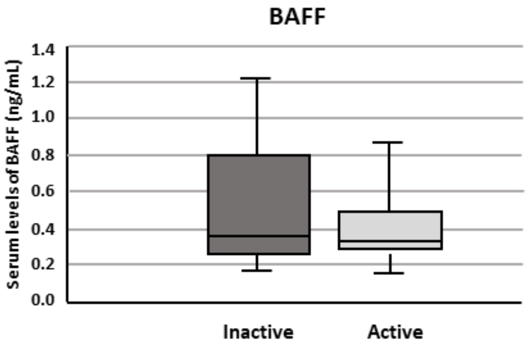

SI Figure 5a

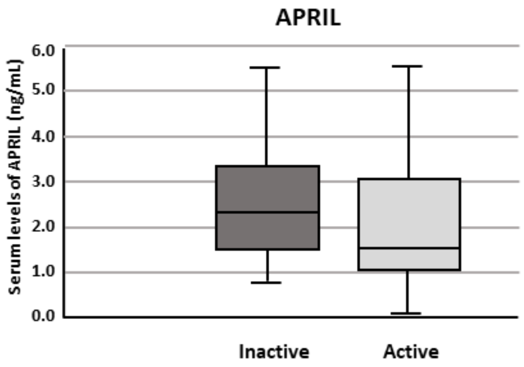

SI Figure 5b

Median levels of sBAFF in patients with an active 0.33, IQR 0.39 and an inactive disease 0.34, IQR 0.32; $p>0.05$

Median levels of sAPRIL in patients with an active 2.21, IQR 1.44 and an inactive disease sAPRIL 1.78, IQR 2.12; $p>0.05$

SI Figure 6. Number of variants in each gene and classification according to their MAF

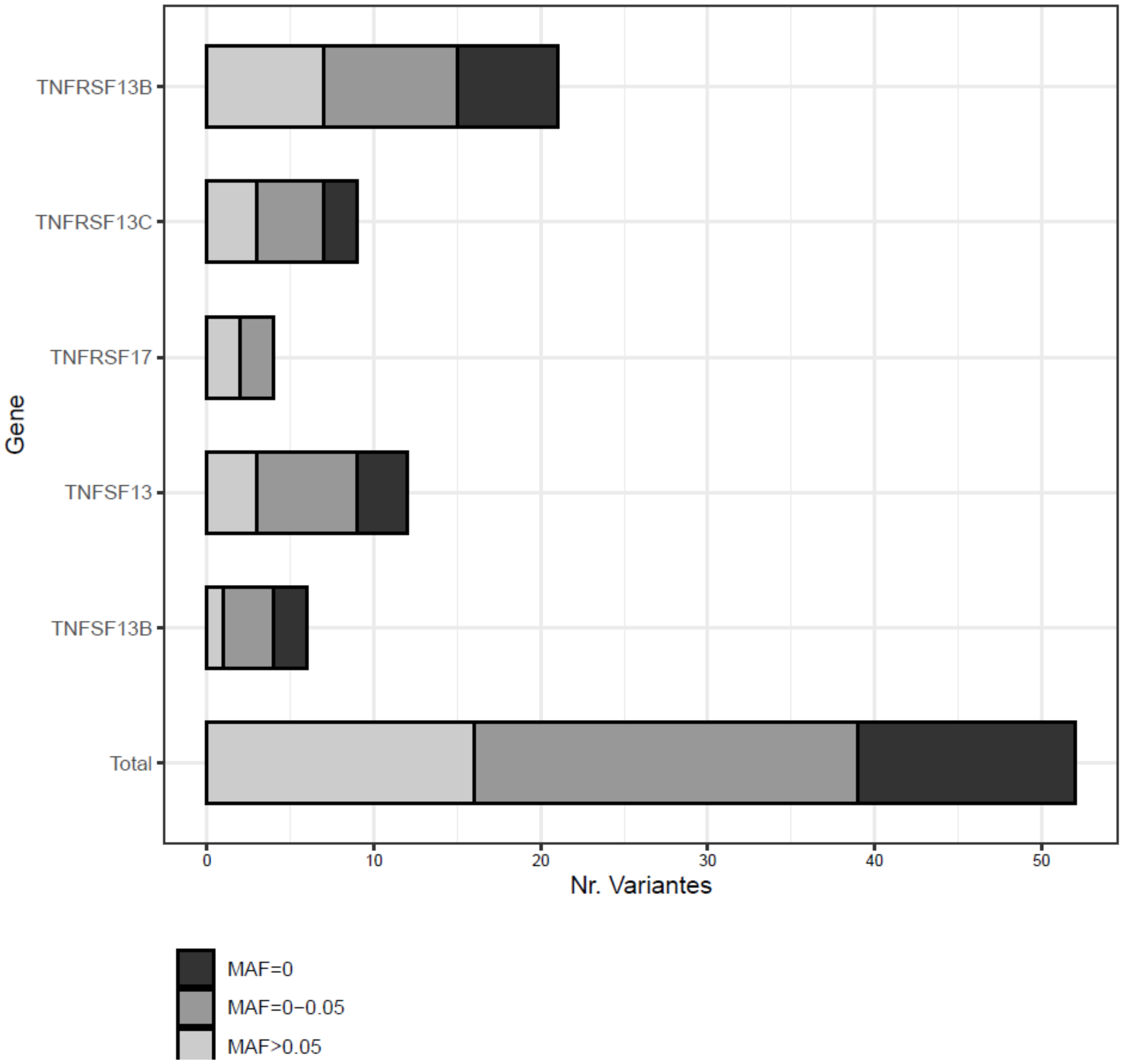

Supplementary information.

SI Table 1. List and description of the genetic variants found in the study

VARIANT	ID	TYPE	ALLELES PATIENTS	ALLELES CONTROLS	MAF (1KG ALL)	MAF (1KG IBS)	ACMG CLASSIFICATION
TNFSF13B_SER42PHE	rs145253799	SNV	1	0	0.0031948881	NA	Likely Bening
TNFSF13B_c.340-45C>G	rs56124946	SNV	8	3	0.069488816	0.028037382	Bening
TNFSF13B_THR143ALA	rs752513510	SNV	0	1	NA	NA	VUS
TNFSF13B_c.482-26C>T	rs73611024	SNV	3	0	0.02755591	NA	VUS
TNFSF13B_ARG231ARG	-	SNV	0	1	NA	NA	Likely Bening
TNFSF13B_c.746-61A>C	rs61972017	SNV	4	3	0.0075878594	0.046728972	Likely Bening
TNFRSF17_ASN81SER	rs373496	SNV	176	171	0.961262	0.9953271	Bening
TNFRSF17_c.277+103G>A	rs11570150	SNV	2	2	0.0041932906	0.009345794	Likely Bening
TNFRSF17_THR159THR	rs2017662	SNV	11	13	0.13977636	0.046728972	Bening
TNFRSF17_THR175THR	rs2071336	SNV	6	9	0.059305113	0.028037382	Bening
TNFRSF13B_c.*175delG	-	INDEL	0	3	NA	NA	VUS
TNFRSF13B_c.*173G>A	rs56153623	SNV	71	66	0.41873002	0.317757	Bening
TNFRSF13B_c.*86_*88delTGA	rs150068036	INDEL	2	6	NA	NA	Bening
TNFRSF13B_SER277SER	rs11078355	SNV	79	77	0.5952476	0.40654206	Bening
TNFRSF13B_THR266ILE	rs752825527	SNV	0	1	NA	NA	VUS-LP
TNFRSF13B_PRO251LEU	rs34562254	SNV	21	15	0.19069488	0.10280374	Bening
TNFRSF13B_VAL220ALA	rs56063729	SNV	0	4	0.0053913738	0.028037382	Bening
TNFRSF13B_c.632-60T>C	rs11652811	SNV	60	60	0.10323483	0.27102804	Bening
TNFRSF13B_ARG202HIS	rs104894649	SNV	1	0	0.00019968051	NA	Likely Pathogenic
TNFRSF13B_c.445+144A>G	rs4517836	SNV	57	56	0.10003994	0.24299066	Bening
TNFRSF13B_c.445+31T>A	rs55955502	SNV	1	1	0.0017971246	NA	VUS
TNFRSF13B_c.445+25A>C	rs2274892	SNV	84	76	0.40634984	0.38317758	VUS
TNFRSF13B_CYS104ARG	rs34557412	SNV	2	4	0.0017971246	NA	Pathogenic
TNFRSF13B_PRO97PRO	rs35062843	SNV	5	7	0.048522364	0.06542056	Bening
TNFRSF13B_ILE87ASN	rs72553877	SNV	2	1	0.00019968051	NA	Likely Pathogenic
TNFRSF13B_ARG72HIS	rs55916807	SNV	0	1	0.0021964856	0.014018691	Likely Bening
TNFRSF13B_LEU69THRfsTer12	rs72553875	INDEL	0	1	NA	NA	Pathogenic
TNFRSF13B_c.200-70C>T	rs537951875	SNV	1	0	NA	NA	VUS
TNFRSF13B_c.200-84_200-83delTT	rs67234667	INDEL	5	8	0.048722044	0.06542056	Bening
TNFRSF13B_MET31THR	rs757910034	SNV	1	0	NA	NA	VUS-LP
TNFRSF13B_THR27THR	rs8072293	SNV	119	118	0.8995607	0.7570093	Likely Bening
TNFSF13_ALA47ALA	rs376426219	SNV	0	1	NA	NA	Likely Bening
TNFSF13_GLY67ARG	rs11552708	SNV	18	6	0.13777955	0.11682243	Bening
TNFSF13_GLY75GLY	rs12942687	SNV	0	1	0.0117811505	0.004672897	Bening
TNFSF13_ASN96SER	rs3803800	SNV	143	142	0.5597045	0.7570093	Bening
TNFSF13_ARG101ARG	rs772960491	SNV	1	0	NA	NA	Likely Bening
TNFSF13_c.337+55G>A	rs72827563	SNV	1	2	0.0029952077	NA	Likely Bening
TNFSF13_c.338-61G>A	rs143094271	SNV	3	3	0.004592652	0.018691588	Likely Bening
TNFSF13_c.505-20_505-18delACA	rs58840546	INDEL	12	11	0.1269968	0.10747664	Bening
TNFSF13_ARG206TRP	rs201011600	SNV	1	0	NA	NA	VUS
TNFSF13_c.*69T>C	rs74875436	SNV	1	0	0.036541533	NA	Bening
TNFSF13_c.*75T>C	rs372917746	SNV	1	0	0.00019968051	NA	Likely Bening
TNFSF13_c.*108G>A	rs6607	SNV	0	2	0.0013977636	NA	Likely Bening
TNFRSF13C_HIS159TYR	rs61756766	SNV	5	1	0.0033945688	0.014018691	Bening
TNFRSF13C_c.368-33T>C	rs5996087	SNV	16	14	0.14496805	0.07009346	VUS
TNFRSF13C_c.367+89G>C	rs73165134	SNV	16	11	0.067492016	0.06542056	VUS
TNFRSF13C_LEU77LEU	rs140820836	SNV	0	1	0.012579872	NA	Bening
TNFRSF13C_GLY64VAL	rs1556157858	MNV	1	0	NA	NA	Likely Bening
TNFRSF13C_c.136+140G>A	rs150150552	SNV	0	3	0.07767572	0.004672897	Bening
TNFRSF13C_c.136+116T>A	rs868540681	SNV	0	1	NA	NA	VUS
TNFRSF13C_PRO21ARG	rs77874543	SNV	13	9	0.044528753	0.06542056	Bening
TNFRSF13C_VAL20VAL	rs373828157	SNV	1	0	0.00019968051	NA	Likely Bening

ACMG American College of Medical Genetics and Genomics

MAF Minor Allele Frequency

VUS variant of uncertain significance

SNV Single Nucleotide Variant

INDEL Insertion/Deletion Variant

MNV MultiNucleotide Variant

IBS Iberian populations in Spain
